# Genotypic Resistance Tests Sequences Reveal the Role of Marginalized Populations in HIV-1 Transmission in Switzerland

**DOI:** 10.1038/srep27580

**Published:** 2016-06-14

**Authors:** Mohaned Shilaih, Alex Marzel, Wan Lin Yang, Alexandra U. Scherrer, Jörg Schüpbach, Jürg Böni, Sabine Yerly, Hans H. Hirsch, Vincent Aubert, Matthias Cavassini, Thomas Klimkait, Pietro L. Vernazza, Enos Bernasconi, Hansjakob Furrer, Huldrych F. Günthard, Roger Kouyos, Swiss HIV Cohort Study, Manuel Battegay, Dominique Braun, Heiner Bucher, Claudine Burton-Jeangros, Alexandra Calmy, Günter Dollenmaier, Matthias Egger, Luigia Elzi, Jan Fehr, Jaque Fellay, Christoph Fux, Meri Gorgievski, David Haerry, Barbara Hasse, Matthias Hoffmann, Irene Hösli, Christian Kahlert, Laurent Kaiser, Olivia Keiser, Helen Kovari, Bruno Ledergerber, Gladys Martinetti, Begoña Martinez de Tejada, Catia Marzolini, Karin Metzner, Nicolas Müller, David Nadal, Dunja Nicca, Giuseppe Pantaleo, Andre Rauch, Stephan Regenass, Christoph Rudin, Franziska Schöni-Affolter, Patrick Schmid, Roberto Speck, Marcel Stöckle, Philip Tarr, Alexandra Trkola, Reiner Weber

**Affiliations:** 1 Division of Infectious Diseases and Hospital Epidemiology, University Hospital Zurich, Zurich, Switzerland; 2 Institute of Medical Virology, University of Zurich, Zurich, Switzerland; 3 Laboratory of Virology, Geneva University Hospital, Geneva, Switzerland; 4 Department of Biomedicine–Petersplatz, University of Basel, Basel, Switzerland; 5 Division of Immunology and Allergy, University Hospital Lausanne, Lausanne, Switzerland; 6 Division of Immunology, University Hospital Lausanne, Lausanne, Switzerland; 7 Division of Infectious Diseases and Hospital Epidemiology, University Hospital Basel, Basel, Switzerland; 8 Division of Infectious Diseases, Cantonal Hospital St. Gallen, St. Gallen, Switzerland; 9 Division of Infectious Diseases, Regional Hospital Lugano, Lugano, Switzerland; 10 Department of Infectious Diseases, Bern University Hospital and University of Bern, Bern, Switzerland; 11 Institute of Social and Preventive Medicine, University of Bern, Bern, Switzerland; 12 Basel Institute for Clinical Epidemiology and Biostatistics, University Hospital Basel, University of Basel, Basel, Switzerland; 13 Kantonsspital Baselland, University of Basel, Basel, Switzerland; 14 Institute of Nursing Science, University of Basel, Basel, Switzerland; 15 University Childrens Hospital, University of Basel, Basel, Switzerland; 16 Department of Sociology, University of Geneva, Geneva, Switzerland; 17 Division of Infectious Diseases, University Hospital Geneva, University of Geneva, Geneva, Switzerland; 18 Global Health Institute, School of Life Sciences, Ecole Polytechnique Fédérale de Lausanne, Lausanne, Switzerland; 19 Clinic for Infectious Diseases and Hospital Hygiene, Kantonsspital Aarau, Aarau, Switzerland; 20 Department of Obstetrics and Gynecology, University Hospital Geneva, University of Geneva, Geneva, Switzerland; 21 University Children's Hospital, University of Zurich, Zurich, Switzerland; 22 Deputy of the patient organization “Positive Council”, Zurich, Switzerland; 23 Division of Infectious Diseases and Hospital Epidemiology, Cantonal Hospital St. Gallen, St Gallen, Switzerland; 24 Childrens Hospital of Eastern Switzerland, St. Gallen, and Division of Infectious Diseases and Hospital Epidemiology, Cantonal Hospital St. Gallen, St Gallen, Switzerland; 25 Centre for Laboratory Medicine, Canton St. Gallen, Switzerland; 26 Cantonal Institute of Microbiology, Bellinzona, Switzerland; 27 Institute for Infectious Diseases, University of Bern, Bern, Switzerland

## Abstract

Targeting hard-to-reach/marginalized populations is essential for preventing HIV-transmission. A unique opportunity to identify such populations in Switzerland is provided by a database of all genotypic-resistance-tests from Switzerland, including both sequences from the Swiss HIV Cohort Study (SHCS) and non-cohort sequences. A phylogenetic tree was built using 11,127 SHCS and 2,875 Swiss non-SHCS sequences. Demographics were imputed for non-SHCS patients using a phylogenetic proximity approach. Factors associated with non-cohort outbreaks were determined using logistic regression. Non-B subtype (univariable odds-ratio (OR): 1.9; 95% confidence interval (CI): 1.8–2.1), female gender (OR: 1.6; 95% CI: 1.4–1.7), black ethnicity (OR: 1.9; 95% CI: 1.7–2.1) and heterosexual transmission group (OR:1.8; 95% CI: 1.6–2.0), were all associated with underrepresentation in the SHCS. We found 344 purely non-SHCS transmission clusters, however, these outbreaks were small (median 2, maximum 7 patients) with a strong overlap with the SHCS’. 65% of non-SHCS sequences were part of clusters composed of >= 50% SHCS sequences. Our data suggests that marginalized-populations are underrepresented in the SHCS. However, the limited size of outbreaks among non-SHCS patients in-care implies that no major HIV outbreak in Switzerland was missed by the SHCS surveillance. This study demonstrates the potential of sequence data to assess and extend the scope of infectious-disease surveillance.

One of the key challenges in HIV surveillance and more generally in infectious-disease epidemiology is that the sampled or surveyed population might not be representative of the target population, especially with respect to hard-to-reach/marginalized subgroups^1^,^2^. The Swiss HIV Cohort Study (SHCS) is one of the most comprehensive HIV cohorts, with an estimated coverage of at least 45% of the cumulative number of HIV-infected individuals in Switzerland, approximately 75% of HIV-patients on antiretroviral treatment, and as much as 80% of AIDS cases^3^. However, since enrolment into the SHCS is voluntary, the possibility remains that marginalized populations are underrepresented and that entire sub-epidemics might be missed by the cohort^2^,^4^.

These issues are particularly important in the context of late presentation of HIV patients, which is a known public health challenge worldwide. Marginalized populations have been shown to be prone to present late as demonstrated in the UK^5^ and Europe in general^6^. Late presentation is one of the hurdles against “treatment-as-prevention” efforts on the population scale^7^. Therefore, marginalized populations present a prime target for intervention and knowing their characteristics would help tailor better interventions^8^.

A unique opportunity to assess the presence of such “under-the-radar” populations is provided by genotypic resistance testing, which is universal in most industrialized countries (i.e. virtually every patient in care receives a resistance test). In our case, we use for Switzerland a database of all genotypic resistance tests (GRT) performed in Switzerland. This database includes both cohort and non-cohort patients and has been initiated by the Swiss-Federal-Social-Insurance Office (“BSV”) as a quality-control measure for HIV genotyping. All Swiss Laboratories which perform routine HIV resistance tests are obliged to submit sequence data along with minimal demographic information for patient de-duplication, into this database, which therefore contains also sequence data for patients that are not enrolled in the SHCS.

This uniquely complete sequence database allows for the assessment of how well the Swiss HIV epidemic is covered by the SHCS. Both an overall good representation and occasional representation gaps appear plausible for this setting: On the one hand, the very high coverage and systematic long-term follow-up of the SHCS argues for a very high representativeness; on the other hand, populations that fear deportation, persecution, or social rejection might be less willing to participate in cohorts^9^.

Molecular phylodynamics^10^ has been used extensively in the recent years to evaluate several features of epidemics^11^, with HIV being one of the more studied viruses in Switzerland^12^ Europe^13^, and the rest of the world^14^. We aim to further investigate the utility of sequence data (which is being rapidly accumulated worldwide) in extending the scope of epidemiological analysis and to assess the unavoidable gaps arising in classical epidemiological surveillance with a focus on marginalized populations (in this study: all patients facing barriers for enrolment). We leverage the versatile tool of molecular phylodynamics to examine whether cohort and non-cohort patients differ with respect to demographics, whether non-cohort patients constitute entire missed outbreaks, and whether missing demographic information can be inferred from clustering patterns.

## Results

We obtained 4,294 non-SHCS HIV sequences from the BSV database collected between 2003 and 2014, of those, 879 were deemed potential duplicates of SHCS patients and thus discarded. Of the remaining 3415, only the first sequence per patient was retained using the workflow delineated in the methods, for a total of 2875 non-SHCS sequences.

The resulting 2,875 non-SHCS sequences were added to the first sequence from the 11,127 SHCS patients with available sequences (out of 18,688 SHCS enrolled patients as of December 2014) for a total of 14,002 Swiss sequences (Table 1). These sequences were then used along with >27,000 background sequences (from the Los Alamos HIV sequence database, see methods) to construct a maximum likelihood phylogenetic tree.

### Demographics analysis

#### SHCS collected demographics

**Figure 1:**
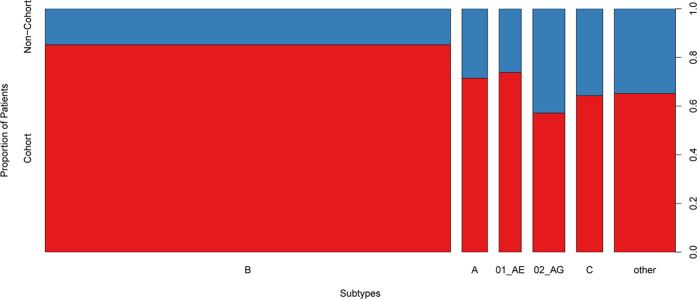
Subtypes Distribution in the overall Swiss patients analysed. The X-axis represents the proportion of the patients, while the breadth of the column reflects the number of Swiss patients per subtype.

We found that SHCS patients differ from non-cohort patients (with a sequence, hereafter referred to as non-cohort or non-SHCS for simplicity) in terms of viral subtype and demographics (Tables 1 and 2). SHCS patients were 29% females, compared to 36% in the non-SHCS. Thus, women were overrepresented among non-SHCS patients (univariable odds ratio (OR) 1.6; 95% confidence interval (CI) 1.4, 1.7),). In regards to subtypes (Fig. 1), univariable analysis showed that non-B subtypes were overrepresented among non-SHCS patients (OR 1.9, 95% CI 1.8, 2.1).

#### Sequence based demographics inference

In order to determine transmission-group and ethnicity for non-cohort patients, we utilized phylogenetic clustering with cohort patients (for whom these demographic variables are known, see methods). This approach is based on the fact that patients in the same phylogenetic cluster tend to exhibit similar demography^15^. The method was validated by testing its predictive power on two subsets of cohort-sequences with known demographics (a random sample from the SHCS and a group of late enrollers, see methods).

Overall, the inference method performed well (up to 81% correct predictions), yet the performance was dependent on the demographic feature predicted and the genetic distance (i.e. the comprehensiveness of sampling and the consequent tightness of social clusters). More complex models (Support vector machines) improved prediction by 3–6%, when taking into account the inferred demographic, the larger cluster demographics distribution, cluster size, and overall genetic distance (results not shown).

The inferred demographics of the non-SHCS individuals were then compared to that of the SHCS (Table 2). In univariable analysis, we found an overrepresentation of heterosexuals (HET) (OR 1.80, 95% CI 1.63, 1.99; compared to MSM), and intravenous drug users (IDU) (OR 1.8, 95% CI 1.52, 2.1) in the non-SHCS, while other transmission-groups were not significantly different between the two populations. Black ethnicity was overrepresented among non-SHCS patients (OR 1.9, 95% CI 1.7, 2.1), while other ethnicities showed no difference. This finding is consistent with the above observation of non-B subtypes being more frequent in non-SHCS patients than in the SHCS, given that non-B subtypes are highly correlated with non-white ethnicities. All the covariates were similar in magnitude and significance in a multivariate model as well (Table 2).

Overall, these analyses reveal a robust overrepresentation of non-B (not imputed) subtypes and non-MSM transmission groups (imputed) among patients not enrolled in the cohort.

### Clusters Analysis

To shed light on possible clusters of patients that are not enrolled in the SHCS we focused on clusters that were predominantly Swiss (>=80% SHCS and non-SHCS) without imposing bootstrap-support or genetic-distance criteria^12^. As previously suggested, such transmission clusters can be interpreted as separate introductions of HIV-1 into Switzerland^12^. Alternative cluster definitions exhibited similar patterns (data not shown).

#### Cluster size distribution and clustering pattern comparison

We found a similar degree of clustering among SHCS and non-SHCS Swiss patients and a strong intermixing between the two populations. 1,645 clusters were composed of >= 80% Swiss sequences, encompassing 8,135 (73%) of the SHCS sequences, and 1,938 (67%) non-SHCS sequences. The median cluster size was 2 with an interquartile range between two and five (Figure S1).

Most non-SHCS patients belonged to mixed clusters (of SHCS and non-SHCS sequences) indicating a strong mixing between SHCS and non-SHCS patients. Specifically, 65% of clustered non-SHCS patients (1253) were part of outbreaks that consisted of >= 50% SHCS sequences (this threshold was chosen to account for the fact that the median cluster size is two).

#### Factors determining predominantly Swiss clusters

Univariable analyses revealed that non-SHCS Swiss sequences were less likely to be part of a Swiss transmission cluster in comparison to SHCS sequences (OR 0.8, 95% CI 0.7, 0.9) however this association was not robust for adjustment (OR 1.0, 95% CI 0.9, 1.1). In both univariable and multivariable analysis, non-B subtype significantly decreased the chances of being in a cluster (OR 0.27, 95% CI 0.25, 0.29), which was similarly reflected in black ethnicity and other non-white ethnicities having similarly lower odds of clustering. The only consistent association of transmission group was that IDUs were more likely to cluster than other transmission groups, while HETs and other transmission groups showed no significant associations (all compared to MSM) (Table 3).

#### Characteristics of purely non-SHCS clusters

**Figure 2:**
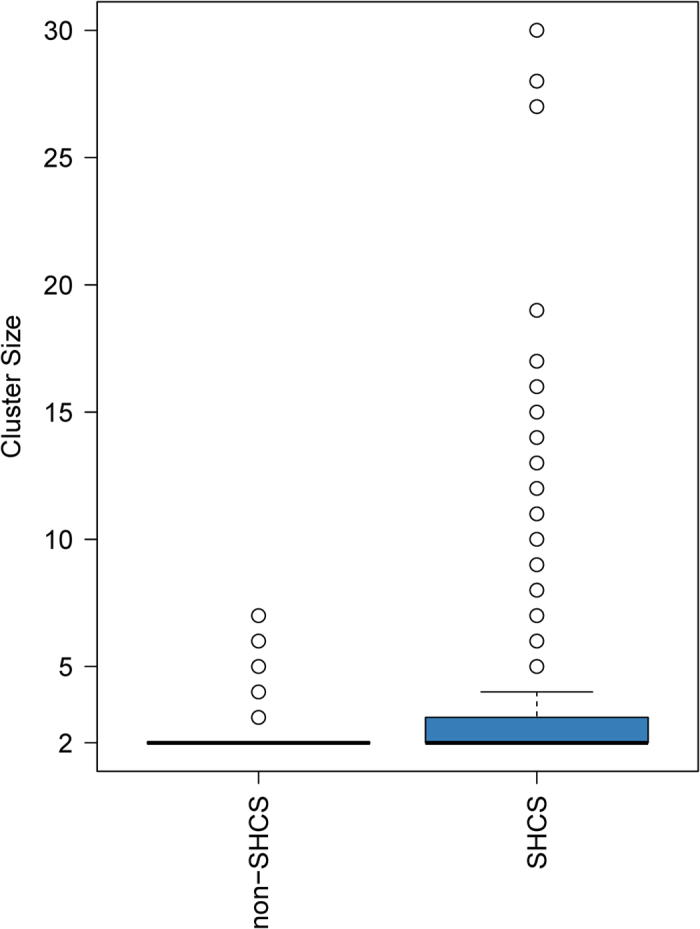
A boxplot of the size distribution of pure SHCS clusters (transmission clusters consisting only of SHCS sequences) and pure non-SHCS clusters SHCS (transmission clusters consisting only of Swiss non-SHCS sequences).

Importantly, we found 344 transmission clusters consisting only of non-SHCS patients. These clusters occurred frequently but were limited in size (Fig. 2) with a median size of 2 patients, IQR: 2–2 and a maximum cluster of seven patients, thus suggesting a strong overlap between transmission chains among SHCS and non-SHCS populations. In particular, this implies the absence of long transmission chains in Switzerland occurring completely outside the SHCS. Finally, there were no apparent subtypes or demographical factors driving the non-SHCS outbreaks (Table 4).

## Discussion

In this study we used a database containing all GRTs performed in Switzerland (since 2003) to evaluate the coverage-especially systematic gaps in the coverage-of the SHCS. Despite having an overall excellent coverage (79% of GRTs were from SHCS patients), we found that non-SHCS patients deviate in terms of demographics in comparison to SHCS patients. The most significant of these differences was an overrepresentation of non-B subtypes among non-SHCS patients. As non-B subtypes dominate in Africa and east Asia^16^, and as these subtypes exhibit a low degree of clustering in our data, this suggests that patients who acquired the infection abroad or from persons originating from those geographical regions, are less likely to enroll in the cohort. Accordingly, we also found that black ethnicity was more frequent among non-SHCS patients. These differences in subtype distribution and ethnicity indicate that hard-to-reach, marginalized populations, or individuals infected abroad were less likely to enrol in the SHCS compared to Swiss or individuals infected in Switzerland. Our results also emphasises the deep intermingling between the two populations, as 65% of non-SHCS patients were within SHCS outbreaks. In summary, non-SHCS patients do not seem to play a significant role in sustaining HIV transmission outside of the cohort, but have significant contributions toward the propagation of non-B subtypes in Switzerland.

As part of the growing understanding that migrants and refugees, especially from HIV-endemic countries are a vulnerable population with specific health needs, the Swiss Federal Office of Public Health is currently conducting a survey among Sub-Saharan African population in Switzerland to map and characterize the needs and attitudes of this population (http://afric-answer.weebly.com/, accessed 1/02/2016). Our work further emphasizes that stigmatization, criminalization, and racial discrimination of patients with HIV are potential obstacles in the battle against the HIV pandemic^9^.

Our sequence-based approach also validated previous independently-derived results about the representativeness of the SHCS^3^,^17^. Our finding that overall 79% of patients with a GRT are enrolled in the SHCS, agrees with previous studies^3^ showing that approximately 75% of patients on ART in Switzerland are in the SHCS. Furthermore, we also found an underrepresentation of non-B subtypes, which is in good qualitative and quantitative agreement with the results from previous studies who also found that migrants were less likely to enrol in the SHCS^4^,^18^.

Despite the differences in the demographics and subtypes, there was no evidence of large outbreaks consisting of purely non-cohort patients (we found a single maximal cluster of 7 patients) (Fig. 2). Theoretically, one cannot rule out that the absence of larger clusters is caused by systematic sample collection bias (for GRTs) that only captures far linked parts of transmission chains, thus leading to smaller observed clusters^19^. However, this is highly unlikely given the universal coverage of the Swiss Health Care System, the fact that genotypic drug resistance testing is part of the standard of care, and the high probability that patients will seek care when they reach the AIDS phase (or often earlier). Furthermore, even for subtype CRF02_AG, the subtype with the worst representativeness, 57% of patients with GRT are enrolled in the SHCS (Table 1), and non-SHCS clusters are characterized by similar demographics than Swiss-transmission clusters in general. Altogether, this indicates that the sampling gaps of the SHCS are not substantial and even sub-epidemics containing non-enrollers are covered (at the very least, partly).

It should be noted that not all HIV positive patients have a genotypic HIV resistance test; thus, there may be fraction of diagnosed patients that are not part of the BSV database and hence not in our study population. For example, there may be a tendency to obtain resistance testing preferentially for patients that are considered eligible for treatment because of low CD4 cell counts, or pregnancy. However, it is likely that such effects have decreased in recent years, as GRTs close to the time of diagnosis have become the standard of care in Switzerland since approximately 2003. In addition, a large part of non-SHCS patients is also cared for in the SHCS centres, thus the same high medical standards are in place for this population and thus no bias towards less resistance testing is expected^4^. Thus the population of patients with a GRT corresponds closely to the population of HIV-patients in care and especially to the population of patients on ART.

We employed a BLAST-based method to retrieve background sequences. The study’s outcomes were unchanged compared to including the entire Los Alamos *pol* sequences (results not shown). Using fewer sequences allowed for smaller trees that take less computational time to be built, thus enabling running the analysis several times as new sequences are added to the SHCS drug resistance database.

In this study, we utilized a liberal cluster definition (80% Swiss with no bootstrap support or genetic distance limitations), which allowed us to capture the maximal number of outbreaks from non-SHCS patients. For the purpose of this study (i.e. excluding large outbreaks among non-cohort patients), this cluster definition is conservative as it treats all possible clusters as true ones. Therefore this approach has the maximal possible sensitivity in detecting outbreaks outside the SHCS. Moreover, our findings remained robust under alternative cluster definitions (combinations of bootstrap support values (70%, 95%) and genetic distances (1.5% and 4.5%). The non-SHCS clusters found were comparable to those found under the presented cluster definition. The SHCS IQR and maximum cluster size remained stable at all combinations strict and liberal, reflecting the robustness of those clusters. Thus despite the lack of a consensus for cluster definitions in molecular epidemiology^20^,^21^,^22^, our findings are robust in this regard.

We also presented a sequence-based method for inferring demographics information for patients with no such data available. This method allowed for the characterization of the demographics of non-SHCS patients and, more generally, proves to be a potential method for extending the coverage of other cohorts by using sequence data from non-cohort individuals (especially given the ever increasing ubiquity of sequencing data). It should be noted that the general problems associated with imputation (see for example^23^) also apply here. As with all inference methods, the quality of the training set plays a vital role in the prediction performance. In our data, some of the patients had uncertainties about the route of infection HET or IDU (7% of the all patients). This was in return reflected in the predictor performance where MSM membership was better predicted than HET and IDU. In addition, there are limitations to the correspondence of the HIV phylogenetic tree and the true underlying structure of the transmission chain, which might affect the prediction performance^21^,^24^. Despite the imperfect performance of the predictor, and given the difficulties associated with evaluating multinomial classification^25^, the predictor still provides some information about an otherwise completely unlit part of the population. Finally, the imperfect performance of the predictor does not affect the other major results of our analysis (overrepresentation of non-B subtypes among non-cohort patients; no large outbreaks completely outside the cohort). Therefore this method could allow the identification of sub-populations who are underrepresented in cohorts and who may therefore profit from additional recruitment and care efforts.

More generally, this work highlights the utility of molecular epidemiology in extending and testing the scope of classical epidemiological data. As sequencing pathogens from a large and representative number of patient samples becomes increasingly affordable, the situation we have encountered with the SHCS and the Swiss HIV epidemic will become more frequent: such a situation is characterized by high-quality and detailed epidemiological data available for a limited number of patients and sequence data available beyond this group. Our work further demonstrates that in this setting sequence data can be used to approximate some of the missing epidemiological information, to assess how well the available epidemiological data captures the spread of an infectious disease (in particular to test whether a cohort misses entire outbreaks), and to identify marginalized subpopulations.

## Methods

### Ethics statement

Participants in the SHCS provided written informed consent and the SHCS, this study, all associated experimental and non-experimental protocols has been approved and is in accordance with local ethics committees’ guidelines in the respective study centres (*Kantonale Ethik-Kommission Zurich, Basel, Bern, Lugano, St Gallen, Geneva and Lausanne)*. In addition, the *Kantonale Ethik-Kommission* Zurich approved of the present analysis (approval number 29/14).

### Patients data

The SHCS-drug-resistance database is part of the SHCS, which is a national cohort study that started in 1988 with ongoing enrolment and semi-annual follow up visits^3^. The SHCS-drug-resistance database contains 21,623 sequences (July 2014) belonging to 11,127 patients. Genotypic resistance tests are done routinely when the patient is first diagnosed, upon a viral-load test, or if virus rebound is observed during ART; in addition retrospective sequencing from the SHCS bio-bank was performed to maximize representativeness. Only the earliest sequence of every SHCS patient was included in the phylogenetic tree, and only from patients belonging to MSM, HET, and IDU transmission groups (covering 96% of HIV cases in the SHCS). Further information about the sequences and their availability can be found in the supplementary material.

The BSV database started collecting genotypic resistance tests in 2003 as they became part of the standard of care in Switzerland; the analysis was hence restricted to data collected after 2003 from the SHCS as well. As some non-cohort patients choose to partake in the SHCS after having been tested and deposited in the BSV-database, suspected duplicate records between non-SHCS and the SHCS were discarded. A non-SHCS sequence was suspected to be part of the SHCS (and hence discarded) if it matched an SHCS patient record on sex and birthdate, and their sequences formed a monophyletic clade.

It is plausible that the same non-SHCS patient might have several sequences in the BSV database, therefore, in order to keep only the single earliest sequence per non-SHCS patient, we assumed that two sequences with the same sex and birthdate and a genetic distance of <2% belonged to the same patient. We choose this conservative cut-off based on Hightower *et al*.^26^ finding that the genetic diversity of HIV pol gene sequence in patients followed longitudinally for a median of 1.8 years was less than 1%, and the median time for SHCS patients between the last negative HIV test and registration being 3.1 years. Technically, a graph was created for every group of sequences sharing the same sex and birthdate with edge-lengths being the genetic distance between two sequences. Edges were dissolved if they carried a genetic distance >2% (indicating that the two vertices/sequences were likely not from the same patient). Isolated vertices were considered unique patients while only the sequence with the earliest sampling date was retained from the connected vertices in the graph.

### Phylogenetic tree construction

To identify transmission clusters, we first pooled the Swiss sequences with non-Swiss sequences from the Los-Alamos sequence database^27^. Specifically, we considered all non-Swiss sequences available in the Los-Alamos sequence database which spanned the protease and reverse transcriptase genes. Of those, only a single sequence per patient was kept provided that the sequence spanned at least 850 nucleotides of the protease and reverse transcriptase (positions 2253–3870 of the HXB2 reference sequence, 114,609 sequences (April/2014)). After excluding Swiss sequences present in the Los Alamos Database, for every Swiss sequence, the ten closest sequences were retrieved using BLAST (Standalone 2.2.28+^28^). Overall, 27,803 foreign sequences were pooled with the Swiss sequences for the final phylogenetic tree construction.

Next, the Stanford and International Antiviral Society-USA drug resistance mutations lists were consulted and the major drug resistance mutations were removed^29^,^30^ to avoid the potentially distorting effect of ART-driven convergent evolution.

Sequences were aligned to HXB2 using Muscle (V3.7^31^, default settings) respectively and the phylogenetic tree was constructed using FastTree 2.1^32^ with the Generalized time-reversible model and the CAT approximation (which has been shown to be better or as accurate as other maximum-likelihood phylogenetic inference methods^33^). Bootstrap-support values for clusters were derived from 100 bootstrap trees (using FastTree 2.1^32^ and GNU Parallel^34^).

### Clusters extraction and demographics prediction

Clusters were defined as monophyletic sub-trees with at least 80% Swiss sequences (SHCS and non-SHCS)^12^. In addition, sensitivity analysis was performed with other cluster definitions (combinations of bootstrap support values (70%, 95%) and inter-cluster genetic distances (1.5 and 4.5%)). Clusters were extracted (using Ape^35^, Caper^36^, R^37^) so that every Swiss sequence could maximally be present once: if a sequence was part of two clusters one of which is nested within the other, only the larger cluster was kept.

For the SHCS sequences, the patients’ demographics were available in the SHCS-database. For Swiss non-SHCS patients, sex and birthdate were provided with the sequences in the BSV-database, other demographic variables (transmission group, ethnicity) were inferred using phylogenetic proximity. Starting from a given non-SHCS tip the tree was traversed up to the parent node then down to the nearest (based on cophenetic distance) SHCS child node with known demographics. If no child node with known demographic were to be found from this node, then the tree was climbed up to the following parent, and the previous process repeated recursively until an SHCS patient with known demographics is found (R code present with supplementary materials). This method was validated on two sets. The first consisted of randomly chosen SHCS patients matching the number of non-SHCS patients (2,875 and for whom demographics was known) with 10-folds cross validation. For the second test set, we approximated the non-SHCS populations by choosing SHCS patients who were found to be HIV-positive at least three years prior to enrolment in the SHCS; those patients were termed “late-enrollers”. As this group also experienced barriers to enrolment in the SHCS, it represents a better approximation for the non-cohort population.

These analyses were performed using uni- and multivariable logistic regression adjusting for the following potential confounders: sex, sample year, subtype, and transmission group.

## Additional Information

**How to cite this article**: Shilaih, M. *et al*. Genotypic Resistance Tests Sequences Reveal the Role of Marginalized Populations in HIV-1 Transmission in Switzerland. *Sci. Rep.* **6**, 27580; doi: 10.1038/srep27580 (2016).

## Supplementary Material

Supplementary Information

## Figures and Tables

**Table 1:** Baseline demographics and the demographics of sequences on the phylogenetic tree stratified by their membership in the SHCS, Switzerland, 1988–2014.

	SHCS	Swiss Sequences (SHCS and non-SHCS)	SHCS sequences	Non-SHCS sequences
Demographics				
Number of Patients	18688	14002	11127	2875
Median Sample year	–	2004 (IQR 1998–2008)	2002 (IQR 1997–2006)	2009 (IQR 2006–2012)
Sex, No. (%)				
	Male 13458 (72%)	9794 (70%)	7956 (72%)	1838 (64%)
	Female 5230 (28%)	4208 (30%)	3171 (29%)	1037 (36%)
Transmission group^b^, No. (%)				
	MSM 6996 (37%)	5252 (38%)	4307 (39%)	945 (33%)
	HET 6153 (33%)	5388 (38%)	3959 (36%)	1429 (50%)
	IDU 4770 (26%)	2769 (20%)	2396 (21%)	373 (13%)
	Other 372 (2%)	246 (2%)	206 (2%)	40 (1%)
	NA 397 (2%)	347 (2%)	262 (2%)	88 (3%)
Ethnicity^b^, No. (%)				
	White 12614 (68%)	10449 (75%)	8623 (78%)	1826 (63%)
	Black 1889 (10%)	2059 (15%)	1318 (12%)	741 (26%)
	Other^c^ 931 (5%)	892 (6%)	657 (6%)	235 (8%)
	NA^c^ 3254 (17%)	602 (4%)	532 (5%)	73 (3%)
Subtype, No. (%)				
	A^a^	624 (4%)	446 (4%)	178 (6%)
	B^a^	9907 (71%)	8440 (75%)	1467 (51%)
	C^a^	647 (5%)	416 (4%)	231 (8%)
	01_AE^a^	546 (4%)	403 (4%)	143 (5%)
	02_AG^a^	784 (6%)	448 (4%)	336 (12%)
	Other Subtypes^a^	1494 (10%)	974 (9%)	520 (18%)

Abbreviations: GRT: Genotypic resistance test, MSM: men who have sex with men, HET: heterosexual, IDU: intravenous drug users, NA: not applicable.

^a^Subtypes can only be determined for patients with a GRT.

^b^Transmission group and ethnicity for non-cohort patients were determined by our phylogenetic predictor (see methods) with no restrictions applied on distance and sampling year.

^c^Other ethnicities encompass all non-white and non-black ethnicities (e.g. Asian), while NA refer to patients with no applicable ethnicity information. For non-SHCS “NA” inferred ethnicity refers to patients to whom the closes SHCS patient had no applicable ethnicity information.

**Table 2:** Univariable and Multivariable logistic regression analysis of the non-SHCS demographics compared to the SHCS^a^ (reference), Switzerland, 2003–2014.

Variable	Univariable OR (95% CI)	Multivariable OR (95% CI)^c^
Transmission Group		
MSM	1 (Reference)	
HET	1.80 (1.63, 1.99)	1.40 (1.22, 1.60)
IDU	1.78 (1.52, 2.07)	2.24 (1.90, 2.65)
Other	1.02 (0.70, 1.48)	0.89 (0.59, 1.31)
Subtypes		
Subtype B	1 (Reference)	
Non-B subtypes	1.94 (1.77, 2.13)	1.57 (1.39, 1.77)
Ethnicity		
White	1 (Reference)	
Black	1.90 (1.69, 2.13)	NA^b^
Other	1.11 (0.94, 1.32)	NA^b^
Sex		
Male	1 (Reference)	
Female	1.56 (1.41, 1.73)	1.26 (1.12, 1.42)
Sample Year	1.17 (1.16, 1.19)	1.19 (1.17, 1.21)

Abbreviations: MSM: men who have sex with men, HET: heterosexual, IDU: intravenous drug users, NA: not applicable.

^a^Cohort membership was the dependent variable with being in the SHCS as the base case.

^b^Ethnicity was not included in the multivariate model because of co-linearity with subtype.

^c^Adjusting for potential confounders sex, sample year, subtype, and transmission group.

**Table 3:** Univariable and Multivariable logistic regression analysis of factors associated with clustering of Swiss sequences.

Variable	Univariable OR (95% CI)	Multivariable OR (95% CI)^b^
Cohort Membership (Baseline SHCS)		
SHCS	1 (Reference)	
Non-SHCS	0.79 (0.72, 0.88)	1.02 (0.91, 1.14)
Transmission Group		
MSM	1 (Reference)	
HET	0.41 (0.37, 0.46)	0.92 (0.80, 1.06)
IDU	1.45 (1.19, 1.77)	1.66 (1.35, 2.05)
Other	0.51 (0.36, 0.73)	0.83 (0.57, 1.23)
Subtypes		
Subtype B	1 (Reference)	
Non-B subtypes	0.25 (0.22, 0.27)	0.30 (0.26, 0.34)
Ethnicity		
White	1 (Reference)	
Black	0.26 (0.23, 0.28)	NA^a^
Other	0.46 (0.4, 0.53)	NA^a^
Sex		
Male	1 (Reference)	
Female	0.49 (1.52, 1.78)	0.75 (0.66, 0.85)
Sample Year	0.98 (0.98, 0.99)	0.98 (0.97, 1.00)

Abbreviations: MSM: men who have sex with men, HET: heterosexual, IDU: intravenous drug users, NA: not applicable.

^a^Ethnicity was not included in the multivariate model because of co-linearity with subtype.

^b^Adjusting for: sex, sample year, subtype, and transmission group.

**Table 4:** Univariable and Multivariable logistic regression of Factors affecting The Clustering of Predominantly Non-SHCS Sequences.

Variable	Univariable OR (95% CI)	Multivariable OR (95% CI)^b^
Transmission Group		
MSM	1 (Reference)	
HET	1.11 (0.93, 1.33)	1.10 (0.88,1.39)
IDU	1.34 (1.03,1.73)	1.44 (1.10,1.87)
Other	1.57 (0.80,3.00)	1.61 (0.81,3.09)
Subtypes		
Subtype B	1 (Reference)	
Non-B subtypes	1.06 (0.90,1.25)	1.12 (0.91,1.39)
Ethnicity		
White	1 (Reference)	
Black	1.08 (0.90,1.30)	NA^a^
Other	0.68 (0.50,0.93)	NA^a^
Sex		
Female	1 (Reference)	
Male	1.10 (0.93, 1.30)	1.18 (0.98, 1.42)
Sample Year		
	1.02 (0.99, 1.04)	1.02 (1.00, 1.05)

Abbreviations: MSM: men who have sex with men, HET: heterosexual, IDU: intravenous drug users, NA: not applicable.

^a^Ethnicity was not included in the multivariate model because of co-linearity with subtype.

^b^Adjusting for: sex, sample year, subtype, and transmission group.
