# Supplementary material for: Genotypic Resistance Tests Sequences Reveal the Role of Marginalized Populations in HIV-1 Transmission in Switzerland
Source: Sci Rep. 2016 Jun 14;6:27580. doi: 10.1038/srep27580 (PMC4906345; doi:10.1038/srep27580)
Supplement: Supplementary Information [file srep27580-s1.doc]

**Title: *Genotypic Resistance Tests Sequences Reveal the Role of Marginalized Populations in HIV-1 Transmission in Switzerland***

**Authors full names and affiliations:** Mohaned Shilaih^1,2^**,** Alex Marzel^1,2^, Wan Lin Yang^1^, Alexandra U. Scherrer^1,2^, Jörg Schüpbach^2^, Jürg Böni^2^, Sabine Yerly^3^, Hans H. Hirsch^4^, Vincent Aubert^5^, Matthias Cavassini^6^, Thomas Klimkait^7^, Pietro L. Vernazza^8^, Enos Bernasconi^9^, Hansjakob Furrer^10^, Huldrych F. Günthard^1,2^, Roger Kouyos*^1,2^, and the Swiss HIV Cohort Study

1- Division of Infectious Diseases and Hospital Epidemiology, University Hospital Zurich

2- Institute of Medical Virology, University of Zurich

3- Laboratory of Virology, Geneva University Hospital, Geneva

4- Department of Biomedicine–Petersplatz, University of Basel, Basel

5-Division of Immunology and Allergy, University Hospital Lausanne, Lausanne

6- Division of Immunology, University Hospital Lausanne, Lausanne

7- Division of Infectious Diseases and Hospital Epidemiology, University Hospital Basel, Basel

8- Division of Infectious Diseases, Cantonal Hospital St. Gallen, St. Gallen

9- Division of Infectious Diseases, Regional Hospital Lugano, Lugano

10- Department of Infectious Diseases, Bern University Hospital and University of Bern, Bern

**Corresponding authors: Roger Kouyos, PhD,** University Hospital Zurich**,** Division of Infectious Diseases**,** and Hospital Epidemiology**,** Rämistrasse 100, CH-8091 Zürich, Switzerland**,** Phone +41 44 255 36 10**,** roger.kouyos@usz.ch

**Mohaned Shilaih,** University Hospital Zurich**,** Division of Infectious Diseases**,** and Hospital Epidemiology Rämistrasse 100**,** CH-8091 Zürich, Switzerland**,** Phone +41 44 255 1816, [Mohaned.shilaih@usz.ch](mailto:Mohaned.shilaih@usz.ch)

Page 1: Figure S1

Page 2: Supplementary code for demographic inference

Page 4: Supplementary data


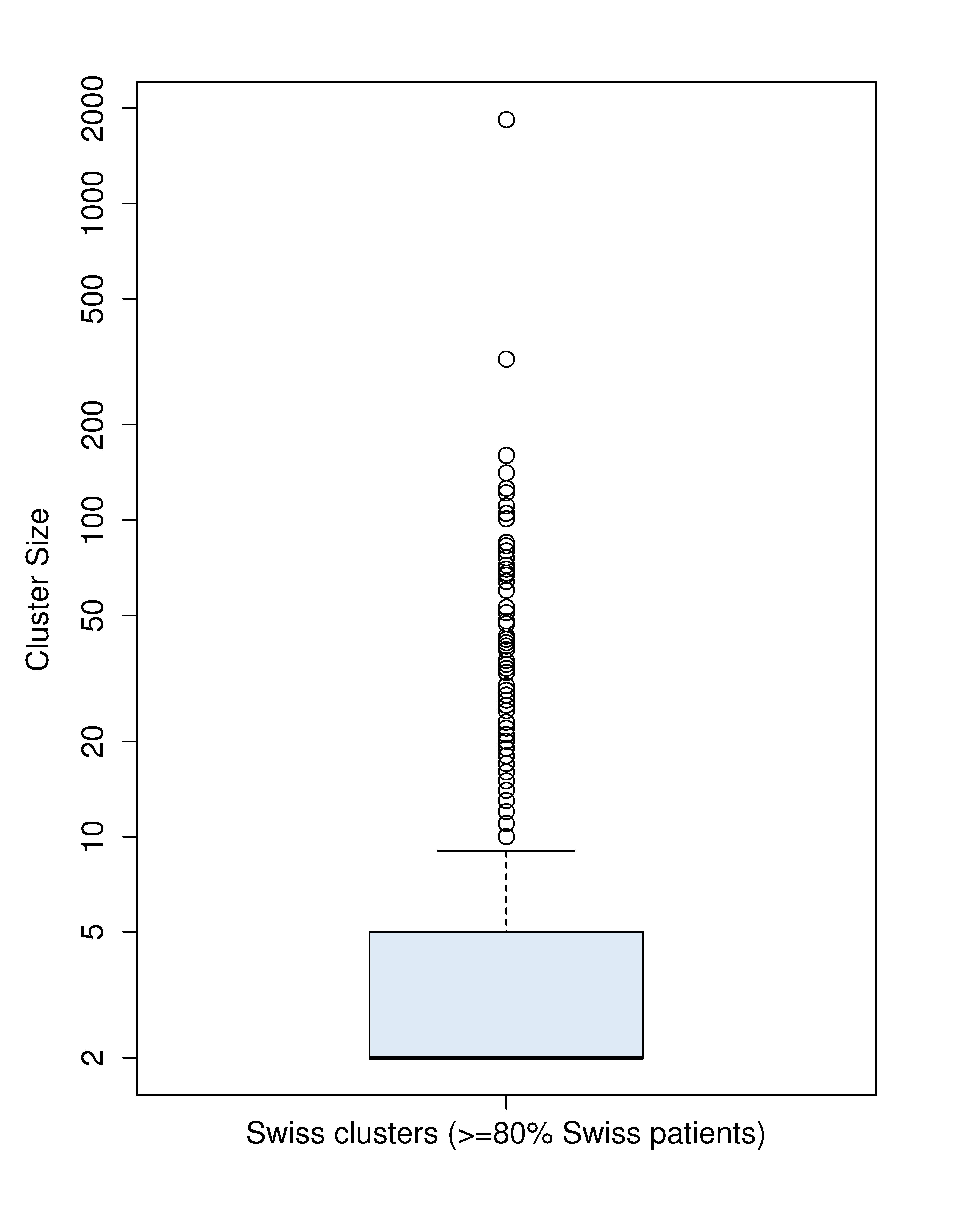


Figure S 1 A box plot of the cluster size distribution of clusters composed mostly of Swiss individuals (>=80%).

#Sample code for demographic inference using phylogenetic proximity

library(ape)

library(caper)

library(e1071)

#read table with test anonymized ids

anonTips = read.table("anonTips")

#read table with known demographics

kDemog = read.table("knownDemog")

#tips names

cTL = as.vector(kDemog$id)

#read in tree built using both sequences

tr = read.tree("fTree.nwk")

closeTips = lapply(anonTips, function(x){

#assumes the tip id corresponding to the sequence id in question

tIndex = which(tr$tip.label %in% x)

#retrieve the parent node of the tip

pNode = edge[as.character(tIndex),1]

#extracts the clade the tip is member of

cld = extract.clade(tr, pNode)

cleanTLabel = cld$tip.label[which(cld$tip.label!=x)]

#make sure at least one sequence with konwn demographic (cTL) is within the clade

while(sum(cleanTLabel %in%cTL)<=1){

#get the parent node of the current node

pNode = edge[as.character(pNode),1]

#get the new clade

cld = extract.clade(tr, pNode)

#TEST PURPOSES ONLY

cleanTLabel = cld$tip.label[which(cld$tip.label!=x)]

}

#gen dist matrix -- different genetic distances could be used here

cpDist = cophenetic.phylo(cld)

#make sure no identical tips are returned

diag(cpDist) = Inf

cNCDist = cpDist[as.character(x),cld$tip.label[which(cld$tip.label %in% cTL)], drop=F]

#retrieve sequence with least distance

return(data.frame(anonTip=x,knownTip=unlist(dimnames(cNCDist[,which.min(cNCDist),drop=F])[2]),dist=min(cNCDist)))

})

#formating

closeTips = as.data.frame(do.call(rbind,closeTips))

#merge first time with konwn demographic data to get true values

clusterTable = merge(closeTips, kDemog, by.x="anonTip",by.y="id", )

#merge another time to get predicted

clusterTable = merge(closeTips, kDemog, by.x="knownTip",by.y="id", suffixes=c(".True",".Pred"))

# test agreement

classAgreement(table(clusterTable$risk.True=="MSM",clusterTable$risk.Pred=="MSM"), match.names=T)

Supplementary data

A sub-sample of over 500 sequences from the SHCS has been uploaded to Genbank (accession numbers, GU344102-GU344671) in the context of a previous publication^1^. In recent study years, the sequences sampling density in Switzerland has reached almost 80% of all patients with an AIDS diagnosis. Due to privacy reasons, the sensitivities associated with HIV infections, and the deep coverage of the dataset, a full deposition of the sequence data in an open database is not possible (Our data would allow for the reconstruction of transmission events and could therefore endanger the patients' privacy). This is exceptionally problematic as HIV-1 sequences have been frequently used in court cases. Finally, all data in the SHCS can be used for well-defined projects that are in accordance with the guidelines of the SHCS, if a corresponding project proposal is submitted to the SHCS scientific board (see www.shcs.ch).

References

1. *Kouyos, R. D. et al. Molecular epidemiology reveals long-term changes in HIV type 1 subtype B transmission in Switzerland. J. Infect. Dis.* ***201,*** *1488–97 (2010).*
